# Occult child abuse presenting as pneumatosis intestinalis and portomesenteric venous gas - a case report

**DOI:** 10.1186/s12887-018-1382-6

**Published:** 2019-01-15

**Authors:** En-Pei Lee, Jainn-Jim Lin, Shao-Hsuan Hsia, Oi-Wa Chan, Han-Ping Wu

**Affiliations:** 1 Division of Pediatric Critical Care Medicine, and Pediatric Neurocritical Care Center, Chang Gung Children’s Hospital and Chang Gung Memorial Hospital, Taoyuan, Taiwan; 2 College of Medicine, Chang Gung University, Taoyuan, Taiwan; 3 Division of Pediatric General Medicine, Chang Gung Children’s Hospital and Chang Gung Memorial Hospital, Taoyuan, Taiwan; 4 Department of Pediatric Emergency Medicine, Children’s Hospital, China Medical University, Taichung, Taiwan; 5 Department of Medicine, School of Medicine, China Medical University, Taichung, Taiwan; 6 Department of Medical Research, Children’s Hospital, China Medical University, Taichung, Taiwan

**Keywords:** Occult child abuse, Pneumatosis intestinalis, Portomesenteric venous gas, Case report

## Abstract

**Background:**

Pneumatosis intestinalis and portomesenteric venous gas are usually caused by necrotizing enterocolitis; however they can occur secondary to abusive abdominal trauma with bone fractures and bruising. It is difficult to recognize initially if there is no bruising on the skin or bone fractures.

**Case presentation:**

We report a 1-year-old child with no obvious history of trauma who presented with conscious disturbance. Abdominal computed tomography showed acute ischemic bowel complicated with pneumatosis intestinalis and portomesenteric venous gas. The first impression was septic shock with acute ischemic bowel. Two weeks after admission, brain magnetic resonance imaging showed subdural hemorrhage of different stages over bilateral fronto-parietal convexities and diffuse axonal injury, suggesting abusive head trauma. He was subsequently diagnosed with occult child abuse.

**Conclusion:**

Pneumatosis intestinalis and portomesenteric venous gas are rare except in cases of prematurity. Occult abusive abdominal trauma should be considered as a differential diagnosis in patients with pneumatosis intestinalis and portomesenteric venous gas, even without any trauma on the skin or bone fractures.

## Background

Pneumatosis intestinalis is the presence of gas in the wall of the intestine, and portomesenteric venous gas is intramural gas drainage into the portal venous system [1]. Both are rare disease entities, and they can sometimes develop simultaneously. They are usually caused by necrotizing enterocolitis, blunt trauma, and mesenteric ischemia [2]. Abusive abdominal trauma in children has also been reported to be a cause of pneumatosis intestinalis and portomesenteric venous gas, when it is accompanied by bone fractures and bruising [3–5]. It is difficult to recognize initially if there is no bruising on the skin or bone fractures. Herein, we report a case of occult child abuse who suffered abusive head trauma and abdominal trauma without any signs of trauma on the skin or bone fractures.

## Case presentation

**Fig. 1:**
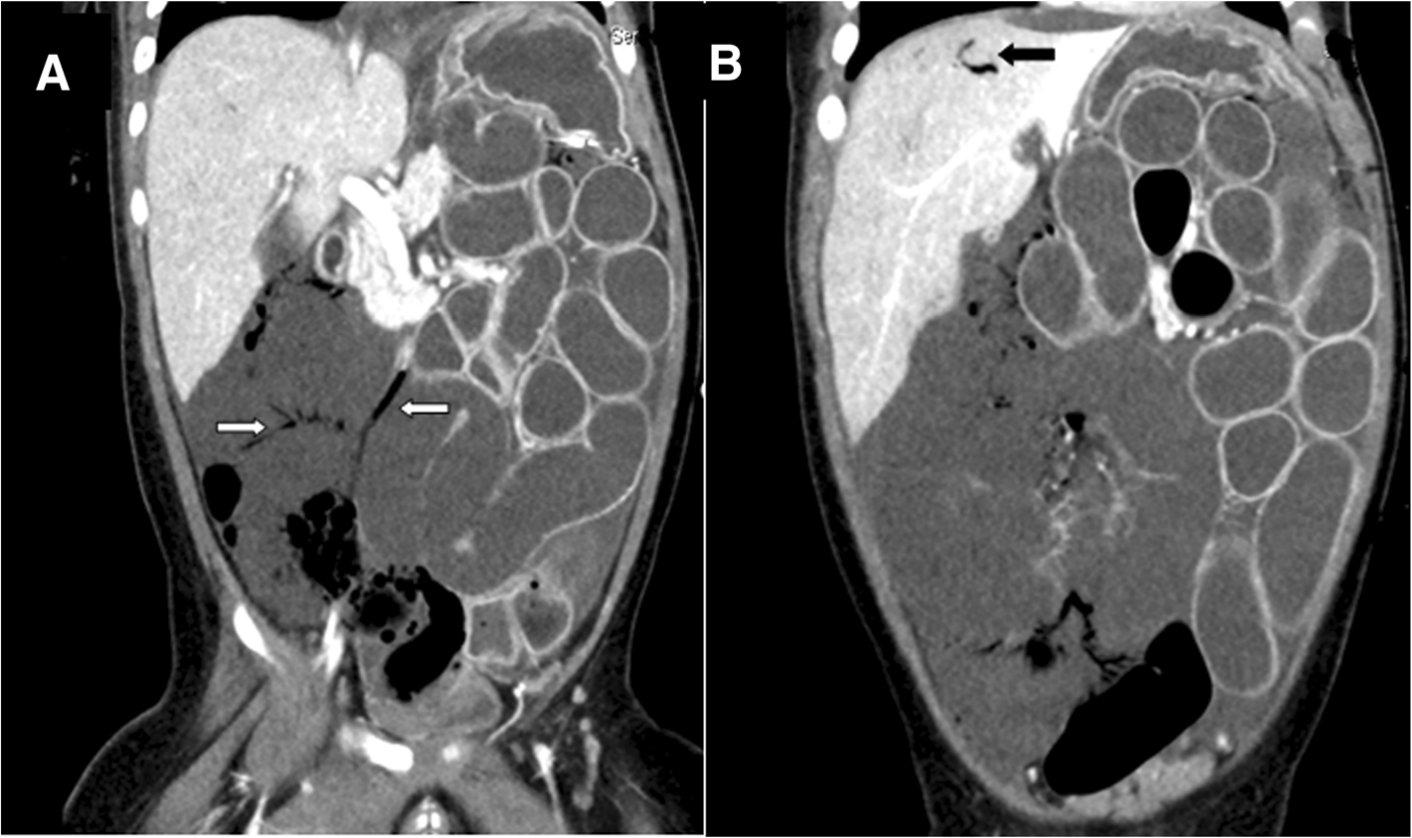
Abdominal CT showed (**a**) intraluminal pneumatosis intestinalis (white arrows) and poor perfusion of the right abdominal bowel wall; (**b**) radiolucent lines in the portal venous system (black arrow)

**Fig. 2:**
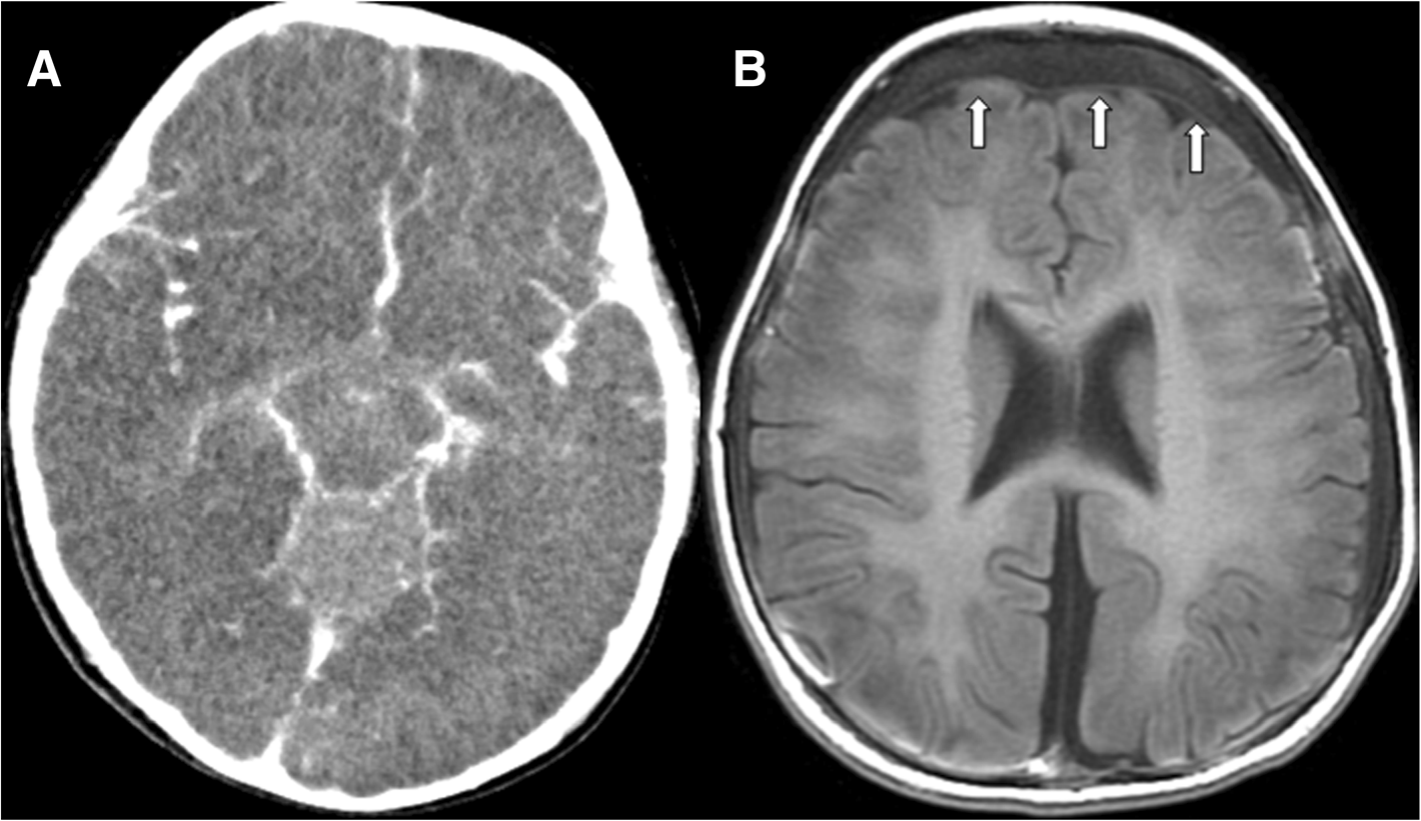
**a**. Brain CT at admission showed hypoxic-ischemic encephalopathy with severe brain edema; (**b**). Brain MRI (T1 flair) at day 14 showed subdural hemorrhage of different stages (white arrows) over bilateral fronto-parietal convexities

A 1-year-old male child was transferred to our emergency room with conscious disturbance. He had no history of fever, upper respiratory tract infection, feeding intolerance, abdominal distension, bloody stools or trauma. His medical history included prematurity, gestational age 29 + 3 weeks with necrotizing enterocolitis stage IA. His parents were young and had a history of drug abuse. The initial Glasgow Coma Scale was E1VEM1 when arrived at our hospital. His vital signs were: temperature, 33.5 °C; pulse rate, 124 beats/min; respiratory rate, 18/min; and blood pressure, 58/47 mmHg. A physical examination showed a distended, guarded abdomen and no obvious bowel sounds. The examination was otherwise unremarkable, and there were no signs of skin bruising or retinal hemorrhage. Laboratory studies revealed a hemoglobin level of 13 g/dl and a white blood cell count of 30,240/ul, with 13% neutrophils. Arterial blood gas analysis showed pH, 6.76; pCO2, 22.5 mmHg; pO2, 97.5 mmHg; HCO3, 3.2 mmHg; and standard base excess, − 31.6 mm/L. Biochemistry studies revealed blood urea nitrogen level, 16 mg/dl; creatinine, 1.07 mg/dl; glutamic pyruvic transaminase, 366 U/l; glutamic oxaloacetic transaminase, 485 U/l; Na, 133 meq/l; K, 6.1 meq/l; and Cl 100 meq/l. A chest X-ray showed diffuse bowel gas with dilatation and suspected ileus. A long bone survey showed no bone fractures. Emergency abdominal computed tomography showed acute ischemic bowel over the right-side of the abdomen, pneumatosis intestinalis (Fig. 1a) and portal vein gas (Fig. 1b). Brain computed tomography revealed diffuse brain swelling with no subdural or subarachnoid hemorrhage with suspected hypoxic-ischemic changes and severe brain edema (Fig. 2a). Considering the clinical presentation and image findings, the first impression was septic shock with acute ischemic bowel. We then performed emergency laparotomy which revealed poor perfusion from the ileum to cecum with a necrotic patch on the bowel wall, consistent with acute ischemic bowel. Two weeks later when he was under a stable condition, brain magnetic resonance imaging was arranged which showed subdural hemorrhage of different stages over bilateral fronto-parietal convexities and diffuse axonal injury (Fig. 2b). Abusive head trauma and abdominal trauma were then diagnosed. He was given total parenteral nutrition, a course of intravenous antibiotics, and ventilatory support. Because of severe brain stem dysfunction, he died on day 43 after admission.

## Discussion

The patient in this case report initially presented with pneumatosis intestinalis, portomesenteric venous gas, disseminated intravascular coagulation and shock. The clinical presentation and image findings initially impressed septic shock with acute ischemic bowel, however physical abuse was less likely because there was no evidence of retinal hemorrhage, bruising or bone fractures and no evidence of subdural or subarachnoid hemorrhage in computed tomography. After 2 weeks of treatment in the pediatric intensive care unit, brain magnetic resonance imaging showed subdural hemorrhage of different stages over bilateral fronto-parietal convexities and diffuse axonal injury. Abusive head trauma and abdominal trauma were then diagnosed. According to his parents, he had developed the symptoms suddenly without initial symptoms of fever, feeding intolerance, abdominal distension or bloody stools. Sepsis could not explain the clinical course. In cases where the clinical course is unusual with no reasonable explanation, other etiologies of acute ischemic bowel such as abusive abdominal trauma should be considered as a differential diagnosis.

Abusive abdominal trauma is the second leading cause of death in physically abused children, most of which are caused by blunt abdominal trauma [4–6]. The liver and small bowel is the most commonly injured organ. Bruising on the abdomen has been reported in 20% of cases of abusive abdominal trauma, and it is usually accompanied by fractures, burns and head injury [5, 7]. Maguire et al. reviewed 88 studies and concluded that small bowel trauma in a child aged less than 5 years with no history of major trauma represents a strong evidence for abusive abdominal trauma [5]. Pneumatosis intestinalis or portomesenteric venous gas can also be caused by blunt abdominal trauma and physical abuse [3, 8–11]. The pathophysiology of pneumatosis intestinalis is characterized by acute pressure changes with the injury leading to mucosal disruption and separation of intraluminal gas in the bowel wall, and sometimes into the portal vein system [8–11]. Pneumatosis coli is defined as gas within the wall of large intestine. In general, the clinical course and prognosis is better in patient with pneumatosis coli compared to pneumatosis intestinalis, because the range of intraluminal gas is shorter and only locate in large bowel in pneumatosis coli which the mucosal damage is longer and severe from small bowel to large bowel in pneumatosis intestinalis [12].

This case highlights the importance of recognizing the clinical signs and symptoms of pneumatosis intestinalis. The family denied any prodromes, and insisted that the child had been healthy, in contrast with our brain magnetic resonance imaging findings (new and old lesions). The other clues for child abuse in our case were the young age, special needs that increased caregiver burden (prematurity), parental characteristics (young parents and a history of drug abuse). When the medical history given by the caregivers is not compatible with the clinical findings, child abuse should be suspected [13–15].

In Taiwan, the Child Protection Medical Service Demonstration Center (CPMSDC) was established to protect children from physical, emotional ill treatment, sexual abuse, and neglect. Our hospital is one of the CPMSDC. This center is composed of social workers, case managers, psychologists, and medical doctors of pediatrics, ophthalmology, obstetrics and gynecology, neurosurgery, radiology, orthopedics, and psychiatry. The center is dedicated to identify and protect children who have been harmed or are at risk of harm, and whose parents are unable to provide adequate care or protection. These cases have been fully discussed at the meeting organized by CPMSDC monthly.

## Conclusions

Pneumatosis intestinalis and portomesenteric venous gas are very rare except in newborns born at term or premature children, and abusive abdominal trauma should be considered as a differential diagnosis even without any signs of trauma on the skin or bone fractures. Follow up imaging is suggested if initial examinations or imaging cannot clarify the etiology. Finally, clinicians should suspect occult child abuse when the medical history given by the caregivers is not compatible with the clinical findings.
